# Girls' perception of physical environmental factors and transportation: reliability and association with physical activity and active transport to school

**DOI:** 10.1186/1479-5868-3-28

**Published:** 2006-09-14

**Authors:** Kelly R Evenson, Amanda S Birnbaum, Ariane L Bedimo-Rung, James F Sallis, Carolyn C Voorhees, Kimberly Ring, John P Elder

**Affiliations:** 1 Department of Epidemiology, School of Public Health, University of North Carolina – Chapel Hill, Chapel Hill, NC, USA; 2 Department of Public Health, Weill Cornell Medical College, New York, NY, USA; 3 Epidemiology Program, Louisiana State University Health Sciences Center School of Public Health, New Orleans, LA, USA; 4 Department of Psychology, San Diego State University, San Diego, CA, USA; 5 Department of Public and Community Health, University of Maryland – College Park, College Park, MD, USA; 6 Department of Biostatistics, Coordinating Center, University of North Carolina – Chapel Hill, Chapel Hill, NC, USA; 7 Graduate School of Public Health, San Diego State University, San Diego, CA, USA

## Abstract

**Background:**

Preliminary evidence suggests that the physical environment and transportation are associated with youth physical activity levels. Only a few studies have examined the association of physical environmental factors on walking and bicycling to school. Therefore, the purpose of this study was (1) to examine the test-retest reliability of a survey designed for youth to assess perceptions of physical environmental factors (e.g. safety, aesthetics, facilities near the home) and transportation, and (2) to describe the associations of these perceptions with both physical activity and active transport to school.

**Methods:**

Test and retest surveys, administered a median of 12 days later, were conducted with 480 sixth- and eighth-grade girls in or near six U.S. communities. The instrument consisted of 24 questions on safety and aesthetics of the perceived environment and transportation and related facilities. Additionally, girls were asked if they were aware of 14 different recreational facilities offering structured and unstructured activities, and if so, whether they would visit these facilities and the ease with which they could access them. Test-retest reliability was determined using kappa coefficients, overall and separately by grade. Associations with physical activity and active transport to school were examined using mixed model logistic regression (n = 610), adjusting for grade, race/ethnicity, and site.

**Results:**

Item-specific reliabilities for questions assessing perceived safety and aesthetics of the neighborhood ranged from 0.31 to 0.52. Reliabilities of items assessing awareness of and interest in going to the 14 recreational facilities ranged from 0.47 to 0.64. Reliabilities of items assessing transportation ranged from 0.34 to 0.58. Some items on girls' perceptions of perceived safety, aesthetics of the environment, facilities, and transportation were important correlates of physical activity and, in some cases, active transport to school.

**Conclusion:**

This study provides some psychometric support for the use of the questionnaire on physical environmental factors and transportation for studying physical activity and active transport to school among adolescent girls. Further work can continue to improve reliability of these self-report items and examine their association of these factors with objectively measured physical activity.

## Background

The socio-ecologic framework emphasizes the multidimensionality of health behaviors, such as physical activity, which are influenced by individual, interpersonal, organizational, community, public policy, and physical environmental variables [1-3]. A basic principle underlying this framework is that physical environments can influence behavior. Perceived environmental barriers are aspects of the environment that an individual views as a hindrance to being physically active, whereas perceived environmental enablers are aspects of the environment that an individual views as helpful to being physically active [4,5]. Perceived environmental factors are hypothesized to affect physical activity behaviors.

To date, few studies have explored the independent effects of these environmental factors on physical activity and active transport to school among adolescents. A review of correlates of physical activity for youth found this to be an understudied area [6]. There is some evidence that the school physical environment is associated with physical activity of adolescents [7] and that changing the environment and policies at school can favorably affect their physical activity [8]. However, it is not clear whether neighborhood environments are also associated with adolescents' physical activity. The adolescent population is an especially important one to study, because during the middle school period physical activity precipitously declines [9,10]. Because girls are consistently found to be less active than boys [6,11], it is important to explore potential environmental contributors to girls' physical activity. Furthermore, walking and bicycling to school declines with higher grade levels [12], making other forms of physical activity that much more important.

Lack of transportation in and around one's neighborhood and to and from activities after school may also be an important barrier to participation in physical activity [11]. A study of middle school boys and girls found that having parents who transported adolescents to physical activity locations was associated with higher reports of physical activity and that boys were transported to locations for physical activity more often than girls [13]. This study was conducted in southern California, and exploration of this association in other geographic areas is warranted.

Moreover, most studies of youth physical activity have measured only recreational activity, but active transportation to school, such as biking or walking, is a potential source of daily physical activity and could provide health benefits [14]. Studies have begun to document the contribution of active commuting to overall physical activity [15-17], but studies are also needed to identify environmental correlates of active transport to school. The purposes of this study were (1) to examine the test-retest reliability of a survey designed for youth to assess perceptions of physical environmental factors (e.g., safety, aesthetics, facilities near the home) and transportation and (2) to describe the association of these measures with physical activity and active transport to school.

## Methods

### Data Collection

The Trial of Activity in Adolescent Girls (TAAG) study is a multicenter group-randomized trial designed to test an intervention to reduce the usual decline in moderate to vigorous physical activity in middle-school girls [18]. The present study was conducted to provide psychometric properties for the instruments to be used in the TAAG study, which was sponsored by the National Heart, Lung, and Blood Institute. Adolescent girls in the sixth and eighth grades were recruited from schools in or near the six TAAG field centers: Baltimore, MD; Columbia, SC; New Orleans, LA; Minneapolis, MN; San Diego, CA; and Tucson, AZ. Data collectors participated in a centralized training to ensure that standardized procedures, scripts, and protocols were used. Girls were recruited while attending required classes at their respective public schools. All participating schools were public schools, one per site, with a mean total enrollment of 872 students (standard deviation 448). The proportion of students receiving free or reduced price lunch varied across schools, from 31.8% to 89.7% (weighted mean 43.5%, standard deviation 12.8%).

Students completed the self-administered questionnaire at school, supervised by the data collectors during February-April 2002. A standardized introduction to the survey was read, and data collectors were available for questions. Readministration of the survey was conducted approximately 2 weeks later (median 12 days, range 6–24 days), using the same procedures to examine reliability of the instrument. Parents or guardians provided written informed consent prior to survey administration. The girls also provided written assent at the time of survey administration. This study was approved by the Institutional Review Board at each field center and at the coordinating center (University of North Carolina – Chapel Hill).

### Survey Instrument

Due to the lack of a developed written questionnaire appropriate for use with adolescents to assess physical environmental factors and transportation in detail, a new questionnaire was developed. Some of the questions were modified from existing questionnaires designed for adults, such as the Amherst Study parent questionnaire [19], the Neighborhood Environment Walkability Scale [20], and a recreational environmental scale [21]. We chose a priori to analyze each of the items separately. Although we were able to group the items into broad domains, we thought it important to describe the individual items (e.g., physical environmental factors and transportation) and their associations with the outcomes separately. In contrast to attitudinal variables, there was not an expectation that the environmental items grouped together would be interrelated, even though they helped define the same construct.

#### Physical Environmental Factors

Physical environmental factors were assessed under the broad categories of perceived safety, aesthetics, and facilities near the home. For perceived safety of the environment, eight items were asked, with possible response options of disagree a lot, disagree a little, neither agree nor disagree, agree a little, or agree a lot.

1. It is safe to walk or jog in my neighborhood.

2. It is safe to ride a bike in my neighborhood.

3. Walkers and bikers on the streets in my neighborhood can easily be seen by people in their homes.

4. There is so much traffic that it makes it hard to walk in my neighborhood.

5. There is a lot of crime in my neighborhood.

6. I often see other girls or boys playing outdoors in my neighborhood.

7. There are lots of loose or scary dogs in my neighborhood.

8. My neighborhood streets are well lit at night.

For aesthetics of the environment, four items were asked with the same response options as above:

1. There are trees along the streets in my neighborhood.

2. There are many interesting things to look at while walking in my neighborhood.

3. When walking in my neighborhood, there are a lot of exhaust fumes or other bad smells.

4. There usually is not garbage or litter in my neighborhood.

The following questions were asked regarding physical activity facilities near home, with the same response options as above:

1. At home there is enough sports equipment to use for physical activity.

2. There are sidewalks on most of the streets in my neighborhood.

3. There are bicycle or walking trails in my neighborhood.

Girls were also provided a list of 14 facilities and asked: (1) "Do you know where a place like this is near your home?" (yes or no) and (2) "Would you go there?" (yes or no). The listed facilities included the following: basketball court, beach or lake, golf course, health club, martial arts studio, playing field (soccer or softball), park, recreation center or YMCA/YWCA, track, skating rink (ice, roller, or inline), swimming pool, walking, biking, or hiking path or trail, tennis court, and dance or gymnastic club. These were scored by adding up the total number of facilities that the participant knew of near her home (possible score range 0–14). We also created a score that added the total number of facilities to the total number to which they would go (possible score range 0–28). This measure was only used as a categorical variable for statistical modeling and was not treated continuously, as we considered it a general indicator of both the number of type of facilities near their home plus the number that they would go to.

#### Transportation

Parental provision of transportation is one form of support, both physical and social, for youth physical activity and active transport to and from school [13]. The following six questions were asked regarding transportation, with response options of disagree a lot, disagree a little, neither agree nor disagree, agree a little, or agree a lot.

1. There are many places I like to go within easy walking distance of my home.

2. My parents (or guardians) worry about something happening to me if I go somewhere on my own.

3. My parents (or guardians) allow me to walk in our neighborhood on my own.

4. My parents (or guardians) allow me to bike on my own.

5. My parents (or guardians) allow me to take public transportation on my own.

6. It is easy to walk or bike to a transit stop (bus, trolley) from my home.

Girls were also asked the following three questions on after-school transportation to/from activities, with the response options including not at all difficult, somewhat difficult, very difficult, or impossible. We did not explore the association of these *after-school *questions with the dependent variable of active transport *to *school, since it did not conceptually make sense.

1. If you stayed after school for an activity everyday, how difficult would it be for you to get home afterward?

2. If you wanted to do an after-school activity someplace else besides school every day, how difficult would it be to get there?

3. If you wanted to do an after-school activity someplace else besides school everyday, how difficult would it be for you to get home afterward?

#### Physical Activity

The Physical Activity Questionnaire for Older Children [22-24], which has been shown to have acceptable reliability and validity when compared to objectively measured physical activity, was modified for use in this study. The modified scale included five of the original nine items that assess activity in the past week from physical education classes, during the lunch period, right after school, in the evenings, and on the weekend. Each of the items had five response options. The questionnaire was scored by adding the responses together and calculating the median (score range 1–5), weighting each question equally, with higher scores indicating more physical activity.

#### Active Transport to School

One item, "How many days in the past week did you walk, bike or skate to school?" with response options as follows: none, 1 day, 2–3 days, 4 days, or every day, constituted the active transport to school construct. This question was assessed separately from the physical activity scale.

#### Other Measures

Race/ethnicity was collected by asking the girls to check all categories that applied: Caucasian (white, non-Hispanic), black, Hispanic, Asian/Pacific Islander, American Indian, or other. Age and grade were self-reported.

### Statistical Analysis

To examine test-retest reliability of the questions, percent agreement (calculated as the number of response pairs agreeing exactly divided by the total number of response pairs) as well as unweighted (2-level) and weighted (3–5-level) kappa coefficients for categorical variables were calculated overall and separately by grade. For continuous variables, intraclass correlations (ICCs) were calculated, using PROC MIXED treating the individual as the repeated measure [25]. The ICCs were recalculated treating site as a random effect, which did not meaningfully change the results and therefore they are not presented. As a rough guide, we followed the ratings suggested by Landis and Koch for agreement [26]: 0–0.2 poor, 0.2–0.4 fair, 0.4–0.6 moderate, 0.6–0.8 substantial, and 0.8–1.0 almost perfect. For the composite measures, Cronbach alpha coefficients were calculated to indicate internal consistency of the items comprising the scales (e.g., how well the items related to one another).

Associations with physical activity and active transport to school were examined using mixed model logistic regression to account for dependence in the data among girls attending the same school. The logistic models were calculated with two outcomes: (1) physical activity score split at the median (equal to or above the median versus below) and (2) biking, walking, or skating to school (1–5 days versus no days). All statistical models were adjusted for grade (sixth versus eighth grade), race/ethnicity (white versus nonwhite), and site. Site, which also represented school (because there was only one school per site), was treated as a random effect in the model using five dummy variables (six sites). In order to explore the relative importance of these variables, we selected the statistically significant (p < 0.05) physical environmental and transportation variables (shown in Tables 4 and 5) and checked for collinearity among these variables. The only variable we did not explore was the continuous measure of the number of facilities near their home, since this contributed to the measure that assessed the number of facilities near their home plus the number of facilities they would go to. None of the variables were collinear, using the guide of proportion of variation being greater than 50% between any two covariates. These variables were then added to a full model, also including grade, race/ethnicity, and site, and the physical environmental and transportation variables were dropped one by one until only significant variables (p < 0.05) remained.

Analyses were conducted using SAS version 8.2 (Cary, NC), and all models were fit using PROC MIXED [25]. To estimate odds ratios the GLIMMIX macro was used, which implements a generalized linear mixed model specifying the error distribution as binomial and the link as logit [27]. It should be noted that because of the common occurrence of our outcomes due to the way they were categorized, the odds ratios we present are likely an overestimation of the relative risk [28].

## Results

Overall 610 girls completed a survey at either the first or second administration, and 480 girls completed surveys at both time points. Approximately half were in the sixth grade and half were in the eighth grade (Table 1). Almost half of the girls were white, 19% were black, and 14% were Hispanic. Approximately the same number of girls participated at each of the study sites.

**Table 1:** Socio-demographic information from first survey of participants (n = 610) *

**Factors**	**Number**	%**
**Age**		
10–11 years	173	28.4
12 years	129	22.3
13 years	172	27.1
14–15 years	122	19.6
		
**Grade**		
6th	311	51.0
8th	299	49.0
		
**Race/Ethnicity**		
Caucasian	295	48.8
Black	113	18.7
Hispanic	85	14.1
Asian/Pacific Islander	19	3.1
Other	10	1.7
Multi-racial	21	3.5
		
**Site**		
Tuscon, AZ	107	17.5
San Diego, CA	103	16.9
Baltimore, MD	105	17.2
Minneapolis, MN	105	17.2
Columbia, SC	98	16.1
New Orleans, LA	92	15.1

*Numbers may not add to 100 due to missing values.

**Percent from the first survey completed by participants.

### Test-Retest Reliability

Among the sample of 610 girls, 480 completed both test and retest surveys (Table 2). Item-specific reliability for eight items assessing safety of the environment ranged from 0.37 to 0.52. Four items assessing aesthetics of the environment ranged from 0.31 to 0.39. Three items assessing facilities near home ranged from 0.42 to 0.58. The reliability of awareness of and interest in going to the 14 recreational facilities ranged from 0.47 to 0.64. The median total number of facilities near one's home was 8 (interquartile range 6–10), and the reliability ICC was 0.78. The alpha coefficient for this score was 0.74 overall, 0.78 for sixth-graders, and 0.70 for eighth-graders. The median total number of facilities near one's home plus the number of facilities they would go to was 18 (interquartile range 14–21), and the reliability ICC was also 0.78. We examined the individual items that made up these scores and found reliability to be consistent across the 14 activities (Table 3, range 0.47–0.64). The alpha was 0.82 overall, 0.84 for sixth-graders, and 0.79 for eighth-graders. The reliability for the nine items assessing transportation ranged from 0.34 to 0.55.

**Table 2:** Test-retest reliability for physical environmental factors, transportation, and physical activity using percent agree, weighted kappa coefficients, or intraclass correlation coefficients (ICC) with 95% confidence intervals (CI)

	**Overall**			**Grade**					
		
	**(n = 480)**			**6th (n = 252)**			**8th (n = 228)**		
			
**Survey Item(s)**	**% Agree**	**Kappa**	**95% CI**	**% Agree**	**Kappa**	**95% CI**	**% Agree**	**Kappa**	**95% CI**
Safety of Environment									
Safe walk/jog	59	0.52	(0.45, 0.58)	60	0.53	(0.43, 0.62)	57	0.50	(0.41, 0.59)
Safe ride bike	58	0.49	(0.42, 0.56)	60	0.52	(0.42, 0.61)	56	0.45	(0.36, 0.55)
Seen by others	51	0.37	(0.30, 0.44)	49	0.34	(0.23, 0.44)	53	0.40	(0.31, 0.50)
Traffic	48	0.38	(0.31, 0.45)	48	0.36	(0.26, 0.47)	49	0.40	(0.31, 0.49)
Crime in neighborhood	61	0.46	(0.39, 0.53)	61	0.44	(0.33, 0.54)	60	0.48	(0.38, 0.58)
See others playing	51	0.47	(0.41, 0.54)	50	0.45	(0.36, 0.55)	52	0.50	(0.41, 0.59)
Loose dogs	46	0.40	(0.33, 0.46)	43	0.34	(0.25, 0.43)	49	0.45	(0.36, 0.55)
Lighting	46	0.48	(0.42, 0.54)	48	0.49	(0.40, 0.57)	43	0.45	(0.37, 0.54)
									
Aesthetics of Environment									
Trees	45	0.37	(0.31, 0.44)	39	0.31	(0.22, 0.41)	50	0.42	(0.33, 0.51)
Things to look at	44	0.39	(0.33, 0.45)	48	0.44	(0.35, 0.53)	39	0.33	(0.24, 0.42)
Smells	54	0.38	(0.31, 0.45)	54	0.36	(0.26, 0.47)	54	0.39	(0.29, 0.49)
Garbage	40	0.31	(0.24, 0.38)	39	0.32	(0.23, 0.42)	42	0.30	(0.20, 0.40)
									
Facilities Near Home									
Equipment	47	0.42	(0.36, 0.48)	49	0.43	(0.34, 0.51)	44	0.39	(0.30, 0.49)
Sidewalks	59	0.58	(0.52, 0.64)	58	0.55	(0.46, 0.64)	61	0.62	(0.54, 0.71)
Trails	47	0.47	(0.41, 0.53)	43	0.42	(0.33, 0.51)	50	0.51	(0.43, 0.60)
*Number of facilities near home		0.78	(0.74, 0.82)		0.81	(0.75, 0.85)		0.75	(0.69, 0.81)
*Number of facilities near home/go there		0.78	(0.74, 0.82)		0.82	(0.77, 0.86)		0.74	(0.67, 0.80)
									
Transportation									
Destinations	46	0.41	(0.35, 0.48)	46	0.36	(0.27, 0.45)	47	0.46	(0.37, 0.55)
Parents worry	40	0.34	(0.28, 0.41)	36	0.28	(0.19, 0.37)	44	0.40	(0.30, 0.49)
Parents let me walk	54	0.55	(0.49, 0.61)	51	0.52	(0.43, 0.60)	57	0.58	(0.50, 0.66)
Parents let me bike	56	0.52	(0.45, 0.58)	52	0.46	(0.37, 0.55)	60	0.57	(0.48, 0.65)
Parents public transportation	50	0.49	(0.43, 0.55)	50	0.48	(0.39, 0.57)	49	0.49	(0.40, 0.58)
Walk to transit	48	0.48	(0.41, 0.54)	46	0.41	(0.32, 0.51)	51	0.54	(0.46, 0.62)
Get home	62	0.38	(0.31, 0.46)	57	0.30	(0.19, 0.42)	64	0.45	(0.35, 0.55)
Get there	59	0.44	(0.37, 0.51)	59	0.45	(0.35, 0.54)	59	0.43	(0.32, 0.53)
Get home afterwards	56	0.41	(0.34, 0.48)	53	0.39	(0.29, 0.50)	58	0.42	(0.32, 0.52)
									
Physical Activity									
*Physical activity score		0.72	(0.67, 0.76)		0.73	(0.66, 0.78)		0.68	(0.60, 0.74)
Days bike/walked/skated to school	74	0.60	(0.52, 0.67)	77	0.64	(0.55, 0.73)	72	0.55	(0.44, 0.66)

*Indicates that ICC used (so percent agreement not calculated), otherwise weighted kappas are used.

**Table 3:** Percent (from first survey) and reliability of a measure of facilities near home for physical activity, using weighted kappa coefficients with 95% confidence intervals (CI)

	**(n = 596)**	**(n = 480)**	
		
**Facilities Near Home**	Unadjusted %*	**Agreement**	**95% CI**
Basketball		0.57	(0.48, 0.65)
Do not know a place	20.7		
Know a place, not go there	11.6		
Know a place, would go there	67.8		
			
Beach or lake		0.64	(0.57, 0.72)
Do not know a place	62.0		
Know a place, not go there	6.2		
Know a place, would go there	31.8		
			
Golf course		0.57	(0.49, 0.65)
Do not know a place	71.7		
Know a place, not go there	17.6		
Know a place, would go there	10.6		
			
Health club		0.61	(0.53, 0.68)
Do not know a place	57.2		
Know a place, not go there	12.7		
Know a place, would go there	30.2		
			
Martial arts studio		0.55	(0.46, 0.63)
Do not know a place	69.4		
Know a place, not go there	13.0		
Know a place, would go there	17.6		
			
Playing field		0.47	(0.37, 0.57)
Do not know a place	13.8		
Know a place, not go there	11.3		
Know a place, would go there	74.9		
			
Park		0.51	(0.39, 0.63)
Do not know a place	9.8		
Know a place, not go there	4.3		
Know a place, would go there	85.9		
			
Recreation center		0.59	(0.52, 0.66)
Do not know a place	46.7		
Know a place, not go there	9.7		
Know a place, would go there	43.7		
			
Track		0.56	(0.49, 0.63)
Do not know a place	45.0		
Know a place, not go there	10.9		
Know a place, would go there	44.1		
			
Skating rink		0.62	(0.54, 0.69)
Do not know a place	37.8		
Know a place, not go there	3.2		
Know a place, would go there	59.0		
			
Swimming pool		0.57	(0.49, 0.66)
Do not know a place	25.7		
Know a place, not go there	5.0		
Know a place, would go there	69.3		
			
Trail		0.53	(0.45, 0.61)
Do not know a place	41.7		
Know a place, not go there	5.6		
Know a place, would go there	52.7		
			
Tennis court		0.62	(0.56, 0.69)
Do not know a place	45.7		
Know a place, not go there	15.6		
Know a place, would go there	38.7		
			
Dance or gymnastics		0.47	(0.39, 0.55)
Do not know a place	56.6		
Know a place, not go there	8.2		
Know a place, would go there	35.3		

*Percent from the first survey completed by participants.

**Table 4:** Percent (from first survey) and association of physical environmental factors (independent variables) to physical activity and active transport to school; OR = odds ratio, CI = confidence interval; models are adjusted for site, race/ethnicity, and grade

		**Physical Activity Above vs. Below Median (n = 610)**		**Active Transport to School (n = 609)**	
			
**Survey Item**	Unadjusted %**	**OR**	**95% CI**	**OR**	**95% CI**
**Safety of Environment**					
Safe walk/jog					
Agree a little/A lot	69.6	2.14	(1.31, 3.48)*	0.97	(0.58, 1.62)
Neither agree or disagree	14.1	1.50	(0.80, 2.80)	0.81	(0.41, 1.59)
Disagree a little/A lot	16.3	1.00		1.00	
					
Safe ride bike					
Agree a little/A lot	77.4	1.45	(0.85, 2.47)	0.87	(0.49, 1.56)
Neither agree or disagree	10.7	0.81	(0.39, 1.68)	0.77	(0.35, 1.69)
Disagree a little/A lot	11.9	1.00		1.00	
					
Seen by others					
Agree a little/A lot	72.0	1.03	(0.60, 1.76)	0.57	(0.31, 1.00)*
Neither agree or disagree	16.6	1.24	(0.64, 2.38)	0.64	(0.32, 1.28)
Disagree a little/A lot	11.5	1.00		1.00	
					
Traffic					
Disagree a little/A lot	72.6	0.91	(0.56, 1.48)	0.91	(0.53, 1.56)
Neither agree or disagree	12.4	0.73	(0.38, 1.40)	1.51	(0.75, 3.04)
Agree a little/A lot	14.9	1.00		1.00	
					
Crime in neighborhood					
Disagree a little/A lot	67.1	0.99	(0.63, 1.55)	1.01	(0.62, 1.66)
Neither agree or disagree	13.0	1.10	(0.60, 2.03)	1.45	(0.76, 2.79)
Agree a little/A lot	19.9	1.00		1.00	
					
See others playing					
Agree a little/A lot	70.3	1.42	(0.91, 2.22)	0.99	(0.60, 1.64)
Neither agree or disagree	10.6	1.38	(0.71, 2.66)	1.46	(0.71, 2.99)
Disagree a little/A lot	19.1	1.00		1.00	
					
Loose dogs					
Disagree a little/A lot	58.5	1.01	(0.67, 1.51)	1.25	(0.79, 1.98)
Neither agree or disagree	15.1	0.66	(0.38, 1.16)	1.46	(0.79, 2.69)
Agree a little/A lot	26.4	1.00		1.00	
					
Lighting					
Agree a little/A lot	49.5	1.40	(0.95, 2.08)	1.16	(0.75, 1.79)
Neither agree or disagree	18.0	1.02	(0.62, 1.70)	1.26	(0.72, 2.18)
Disagree a little/A lot	32.4	1.00		1.00	
					
**Aesthetics of Environment**					
Trees					
Agree a little/A lot	61.7	1.78	(1.17, 2.72)*	0.96	(0.61, 1.50)
Neither agree or disagree	13.7	1.47	(0.82, 2.64)	0.87	(0.46, 1.66)
Disagree a little/A lot	24.6	1.00		1.00	
					
Things to look at					
Agree a little/A lot	49.2	2.36	(1.56, 3.59)*	1.41	(0.89, 2.24)
Neither agree or disagree	22.9	1.31	(0.81, 2.14)	1.53	(0.89, 2.63)
Disagree a little/A lot	27.9	1.00		1.00	
					
Smells					
Disagree a little/A lot	71.6	1.08	(0.66, 1.75)	0.43	(0.26, 0.71)*
Neither agree or disagree	12.8	0.85	(0.45, 1.63)	0.35	(0.17, 0.72)*
Agree a little/A lot	15.6	1.00		1.00	
					
Garbage					
Agree a little/A lot	51.9	1.78	(1.20, 2.65)*	1.27	(0.82, 1.97)
Neither agree or disagree	16.5	1.18	(0.70, 1.98)	1.39	(0.79, 2.46)
Disagree a little/A lot	31.7	1.00		1.00	
					
**Facilities Near Home**					
Equipment					
Agree a little/A lot	56.4	2.50	(1.65, 3.79)*	1.02	(0.65, 1.61)
Neither agree or disagree	17.9	1.45	(0.85, 2.46)	1.56	(0.89, 2.75)
Disagree a little/A lot	25.7	1.00		1.00	
					
Sidewalks					
Agree a little/A lot	68.4	1.34	(0.89, 2.02)	1.05	(0.66, 1.67)
Neither agree or disagree	7.9	1.43	(0.71, 2.91)	1.29	(0.60, 2.79)
Disagree a little/A lot	23.7	1.00		1.00	
					
Trails					
Agree a little/A lot	47.4	1.68	(1.16, 2.44)*	1.59	(1.05, 2.40)*
Neither agree or disagree	13.8	1.15	(0.68, 1.96)	0.75	(0.39, 1.42)
Disagree a little/A lot	38.8	1.00		1.00	
					
Number of facilities near home					
>=10	37.7	2.26	(1.44, 3.57)*	2.26	(1.36, 3.76)*
>=7–9	31.9	1.56	(0.98, 2.49)	1.46	(0.86, 2.47)
<7	30.4	1.00		1.00	
					
Number of facilities near home/go there					
>=20	34.9	2.75	(1.72, 4.40)*	1.94	(1.17, 3.21)*
16–19	33.1	1.08	(0.68, 1.72)	0.76	(0.44, 1.29)
<16	32.0	1.00		1.00	

*Indicates OR with p < 0.05; **Percent from the first survey completed by participants.

**Table 5:** Percent (from first survey) and association of transportation measures to physical activity and active transport to school; OR = odds ratio, CI = confidence interval; models adjusted for site, race/ethnicity, and grade

		**Physical Activity Above vs. Below Median (n = 610)**		**Active Transport to School (n = 609)**	
			
**Survey Item**	Unadjusted %**	**OR**	**95% CI**	**OR**	**95% CI**
Destinations					
Agree a little/A lot	70.7	1.78	(1.11, 2.83)*	1.44	(0.85, 2.43)
Neither agree or disagree	12.3	1.38	(0.73, 2.61)	1.01	(0.49, 2.08)
Disagree a little/A lot	17.0	1.00		1.00	
					
Parents worry					
Disagree a little/A lot	27.0	1.43	(0.96, 2.14)	0.82	(0.53, 1.28)
Neither agree or disagree	11.5	1.58	(0.91, 2.75)	0.73	(0.39, 1.35)
Agree a little/A lot	61.5	1.00		1.00	
					
Parents let me walk					
Agree a little/A lot	59.6	1.79	(1.18, 2.70)*	1.14	(0.72, 1.79)
Neither agree or disagree	15.6	0.98	(0.56, 1.71)	1.44	(0.80, 2.62)
Disagree a little/A lot	24.8	1.00		1.00	
					
Parents let me bike					
Agree a little/A lot	69.1	1.38	(0.89, 2.13)	1.15	(0.71, 1.87)
Neither agree or disagree	10.6	1.09	(0.57, 2.08)	1.54	(0.76, 3.10)
Disagree a little/A lot	20.4	1.00		1.00	
					
Parents public transportation					
Agree a little/A lot	27.0	1.59	(1.05, 2.41)*	1.36	(0.86, 2.14)
Neither agree or disagree	18.2	1.18	(0.74, 1.88)	1.54	(0.94, 2.53)
Disagree a little/A lot	54.7	1.00		1.00	
					
Walk/bike to transit					
Agree a little/A lot	51.3	1.71	(1.15, 2.55)*	1.90	(1.21, 3.00)*
Neither agree or disagree	15.3	1.39	(0.81, 2.37)	2.16	(1.20, 3.87)*
Disagree a little/A lot	33.5	1.00		1.00	
					
Get home+					
Not at all difficult	3.4	0.73	(0.26, 2.05)		
Somewhat difficult	11.7	0.99	(0.55, 1.78)		
Very difficult	32.0	0.71	(0.48, 1.07)		
Impossible	53.0	1.00			
					
Get there+					
Not at all difficult	5.6	1.31	(0.55, 3.14)		
Somewhat difficult	13.7	0.53	(0.30, 0.94)*		
Very difficult	42.9	0.68	(0.45, 1.02)		
Impossible	37.9	1.00			
					
Get home afterwards+					
Not at all difficult	6.1	1.92	(0.84, 4.37)		
Somewhat difficult	17.2	0.86	(0.51, 1.45)		
Very difficult	35.4	0.83	(0.55, 1.26)		
Impossible	41.3	1.00			

*Indicates OR with p < 0.05; **Percent from the first survey completed by participants.

+These 3 questions regarding after school transportation were not explored with the dependent variable of active transport to school.

The median physical activity score was 3.0 (interquartile range 2.4–3.6, range 1–5). The test-retest reliability of the physical activity score was 0.72 (Table 2), with no meaningful differences by grade. The alpha was 0.61 overall, 0.58 for sixth-graders, and 0.59 for eighth-graders. Overall, 42.3% of girls reported any active transport to school, ranging from 13.3% in Minneapolis to 54.3% in Baltimore. Overall, 4.9% reported 1 day of active transport to school in the past week, 7.2% reported 2–3 days, 2.5% reported 4 days, and 15.1% reported everyday. The test-retest reliability of the number of days of active transport to school was 0.60 with no differences by grade.

### Associations with Physical Activity and Active Transport to School

Tables 4 and 5 display the percent of each physical environmental and transportation items and its corresponding association with physical activity and active transport to school, adjusted for race/ethnicity, grade, and site. Table 6 displays the results of the analysis of the relative importance of each physical environmental and transportation variable. We highlight associations reaching a significance of p < 0.05.

**Table 6:** Models showing association of significant physical environmental factors and transportation (independent variables) to physical activity and active transport to school; OR = odds ratio, CI = confidence interval; models are adjusted for site, race/ethnicity, grade, and other variables listed under each dependent variable

	**Physical Activity Above vs. Below Median (n = 610)**		**Active Transport to School (n = 609)**	
		
**Survey Item**	**OR**	**95% CI**	**OR**	**95% CI**
Things to look at				
Agree a little/A lot	1.91	(1.17, 3.11)*		
Neither agree or disagree	1.05	(0.59, 1.87)		
Disagree a little/A lot	1.00			
				
Smells				
Disagree a little/A lot			0.43	(0.24, 0.75)*
Neither agree or disagree			0.38	(0.18, 0.81)*
Agree a little/A lot			1.00	
				
Equipment				
Agree a little/A lot	2.51	(1.51, 4.17)*		
Neither agree or disagree	1.52	(0.80, 2.86)		
Disagree a little/A lot	1.00			
				
Number of facilities near home/go there				
>=20	1.79	(1.06, 3.03)*	1.92	(1.15, 3.22)*
16–19	0.81	(0.49, 1.33)	0.78	(0.45, 1.34)
<16	1.00		1.00	
				
Walk/bike to transit				
Agree a little/A lot	1.82	(1.14, 2.89)*		
Neither agree or disagree	1.64	(0.88, 3.09)		
Disagree a little/A lot	1.00			

*Indicates OR with p < 0.05.

#### Safety of Environment

Girls who agreed with the statement "it is safe to walk or jog in my neighborhood" were more than twice as likely to report more physical activity (i.e., in the upper half of the distribution) compared to girls who disagreed with this statement (Table 4).

#### Aesthetics of Environment

Girls reporting more trees, interesting things to look at, and lack of garbage or litter in the neighborhood were more likely to report physical activity than girls not reporting these characteristics (Table 4). Reporting interesting things to look at remained associated with physical activity in the overall model (Table 6). Not having bad smells in the neighborhood was associated with a decreased odds of reporting active transport to school (Table 4) and remained in the overall model (Table 6).

#### Facilities Near Home

Girls who reported sports equipment at home were more than twice as likely to report physical activity than girls not having access to equipment (Table 4), which remained in the overall model (Table 6). Girls with bicycle or walking trails in their neighborhood were more likely to report both physical activity and active transport to school than girls without trails. To examine whether the number of activity facilities near a girl's home was associated with physical activity, we categorized the scores into tertiles. Girls who reported more facilities (i.e., highest tertile) were significantly more likely to report physical activity and active transport to school than girls in the lowest tertile. Furthermore, when we accounted for whether girls would go to those facilities, those in the highest tertile were 2.75 times more likely to report physical activity and almost twice as likely to report active transport to school than girls in the lowest tertile. The association between the highest tertile and both physical activity and active transport to school remained in the overall models (Table 6).

#### Transportation

Girls who agreed there were destinations of interest within walking distance of their homes were more likely to report physical activity than girls disagreeing with this statement (Table 5). Furthermore, girls who agreed that parents would let them walk in their neighborhood, take public transportation on their own, or walk/bike to transit were more likely to report physical activity than girls disagreeing with these statements. Being able to easily walk or bike to transit remained in the overall model (Table 6) and was also associated with active transport to school (Table 5). Girls who found it "somewhat difficult" to get to after-school activities were less likely to report physical activity than those who found it "impossible."

## Discussion

This study examined the test-retest reliability of a survey to assess physical environmental factors and transportation, and then examined associations of these factors with physical activity and active transport to school among adolescent girls. Overall, the 24 individual items on safety, aesthetics, facilities near the home, and transportation mostly indicated fair to moderate reliability (kappa range 0.28–0.62) among sixth- and eighth-grade girls, usually without differences across grade. For the two scales on physical activity facilities near the home, reliability measures indicated substantial reliability (ICC range 0.74–0.82). These reliability results for 10–15 year-old girls were generally similar to or higher than reliability reported in other studies of youth [29,30]. Our study identified several cross-sectional associations with the physical environment and transportation measures when controlling for age, race/ethnicity, and site. These are discussed next in the context of the scientific literature.

### Safety of Environment

Neighborhoods with signs of physical disorder (e.g., graffiti, vandalism) and social disorder (e.g., loitering, drug use) may discourage individuals from engaging in outdoor activities [31,32]. Concerns for neighborhood safety may be reported more frequently for girls than boys [11]. Our study found that agreeing it was safe to walk or jog in the neighborhood was associated with higher levels of physical activity. Findings in the literature of the effect of safety on physical activity of youth have been mixed. Although perception of safety was associated with increased physical activity in some studies [33], a lack of association was reported in other works [19,29,34-40] and with decreased physical activity in at least one study [41]. However, studies using objective measures of crime identify unsafe neighborhoods as being associated with less physical activity [42-44]. For example, in a smaller study conducted in Texas, the density of violent crimes within 0.5 mile of the homes of mostly Mexican American seventh-graders was associated with less outdoor physical activity among girls but not boys [42]. In this referenced study, girl's perception of safety was associated with higher levels of outdoor physical activity.

Our study also found a lack of association of most safety variables with active transport to school. We did find that agreeing that "walkers and bikers on the streets in my neighborhood can easily be seen by people in their homes" was associated with less report of active transport to school, rather than more. A 2004 U.S. survey of households with children 5–18 years old indicated that parents reported traffic and crime as barriers to walking and biking to school [45]. Safety has also been reported as an important barrier to children walking and bicycling to school in other U.S. studies [46] and in other countries, such as New Zealand [47], England [48], Canada [49], and Australia [50-54]. In particular, three quantitative studies examined the relationship between environmental factors and active transport to school. First, a study in England [48] found that children of parents who worried about their children becoming lost or being abducted were less likely to walk to school. Second, a study in Australia found that parental perceptions of the neighborhood were associated with walking and cycling to school among 10–12-year-olds [51,54]. Another Australian study of adolescent youth found that walking to school was negatively associated with parental concern about traffic but positively associated with having friends living in the neighborhood for girls but not for boys [52]. The discrepancy between our findings and these reports could be due to the contrast between parental perception, which we did not measure, compared to the perception of youth, which we measured. It may be that parents' perceptions of safety are more important than those of youth in determining active transport to school. In fact, the Australian study [51] found that there were some discrepancies between parental and child report of perceptions of traffic and safety and that indeed the parental perceptions might be more important.

### Aesthetics of Environment

Aesthetic features, such as trees, things to look at (e.g., better scenery), and garbage were not associated with increased walking/cycling to school but were associated with physical activity. This is supported in a study of Portugese youth in 7th-12th grades, where having many interesting things to look at (this question was similarly worded to our question) was a significant positive predictor of physical activity [29]. Furthermore, we found that unpleasant smells in the neighborhood was not associated with overall physical activity but was associated with higher reports of active transport to school. Girls who actively travel to school on foot or by bicycle may be more able to notice odors in the environment than they would if they were traveling by automobile or bus.

### Facilities Near Home

According to a review by Sallis et al. [6], having equipment and supplies available for activity was positively associated with physical activity in only one of five studies of adolescents. We found that girls were more than twice as likely to be more physically active when they reported enough sports equipment at home. Differences between studies may be due to the differing measures of access to fitness/sports equipment, as well as differing measures of physical activity. Some studies may have also lacked sufficient statistical power to detect an association.

An environment rich in physical activity resources could remain underutilized unless consideration is given to how accessible the facilities are to the population being served. Again, according to the review by Sallis et al. [6], having access to facilities and programs for activity was positively associated with physical activity in three of four studies of children 4–12 years of age. Since then, several other studies have examined associations with community facilities. A study of youth in grades 1–12 found no association of a single item measure assessing access to parks, playgrounds, or gyms with objectively measured physical activity [19], whereas in a study of Portugese youth in 7th-12th grades, a single item measure of access to physical activity facilities was associated with self-reported physical activity [29]. In a study of 87 adolescent girls, the number of self-reported community facilities was correlated positively with cardiorespiratory fitness but not with physical activity [55]. In a study of mostly Mexican American seventh-graders, euclidean distance from their home to the nearest public play area (i.e., playgrounds, swimming pools, fields) was associated with outdoor physical activity among boys but not girls [42]. A national U.S. study of 7th-12th graders found that presence of a physical activity facility within a given census block group was associated with self-reported physical activity [56]. Furthermore, a study from Australia found that youth 10–12 years old who perceived poor access to parks in their neighborhood were less likely to walk or cycle to school [51]. However, a prospective study of high school girls did not find an association between a 3-item lack of physical activity resource measure and changes in self-reported physical activity [57].

In the present study, girls who reported more physical activity facilities was associated with higher levels of physical activity and active transport to school. This could be the case since girls who are active and walk and bicycle through their environment may be more aware of their surroundings and thus, the physical activity facilities that exist. This was also similar for girls reporting biking or walking trails in their neighborhood. This finding has important implications for further study. It is not clear whether the girls' perceptions of facilities matched what actually existed in their neighborhoods. Studies comparing objective measures of facilities to perceptions can help further address this.

### Transportation

Having parents who provide transportation, in general or specifically to physical activity destinations, was positively associated with physical activity behaviors in some studies but not all [35,37,58,59]. Several of these studies showed gender differences in this finding [35,58]. Hoefer et al. [13] found that parental transportation was associated with physical activity and that parental transportation may be more important for girls than boys. This was also supported in a national parental survey of youth 9–13 years of age, where transportation problems were reported more often as a barrier for girls than boys [11]. We found that having parents who allowed girls to walk in the neighborhood on their own, use public transportation on their own, or could walk/bike to transit from their home was associated with higher levels of physical activity.

### Limitations

The work reported here provides support for continuing to explore the role of the physical environment and transportation on girls' physical activity and active transport to school. This study is limited by several factors. First, there may have been other important confounders that we did not account for in the adjusted models for which we did not have measures, such as socioeconomic status, number of children in the family, and parents' and siblings' physical activity. Second, our self-report measures are subject to social desirability bias, which may have affected both the independent and dependent variables [60]. Future studies exploring this should consider using objective measures in addition to self-report measures. Third, despite the short time between administrations, true changes in physical activity could have occurred between the two surveys, which would affect our reliability estimates and could affect our ability to assess associations with physical activity and active transport to school [61,62]. If this did occur, the true agreement could be higher than we reported but would also be attenuated due to measurement error. It is unlikely that the neighborhood measures changed in this short time period. Fourth, this study is cross-sectional in design, so the direction of the relationships is not known. Fifth, in this study we have tested many associations, but chose not to adjust for multiple testing because we considered this study exploratory. Therefore, significance should be interpreted with caution and replication of results is needed. Lastly, this study explored factors related to active transport to school, but not active transport from school. Results from qualitative research suggest that in some cases barriers to active travel from school may differ from those going to school [46].

## Conclusion

The present study documented fair to moderate test-retest reliability for most items on the survey of physical environmental factors and transportation among adolescent girls. We found that some items on perceived safety of the environment, aesthetics of the environment, facilities near the home, and transportation appeared to be important correlates of physical activity, and in some cases, active transport to school. Further work can continue to improve reliability of these self-report items, begin to explore validity (such as what has been done with children's ability to report traffic [63]), evaluate additional environmental factors, and examine the association of these factors with objectively measured physical activity. Future studies could examine these associations longitudinally and explore how the environment and transportation might act as mediators or moderators to physical activity and active transport to school. Improved understanding of correlates, mediators, and moderators of girls' physical activity is needed to identify policy and environmental initiatives that hold promise for increasing girls' physical activity levels to provide health benefits.

## Competing interests

The author(s) declare that they have no competing interests.

## Authors' contributions

KRE participated in the development of the questionnaire, analysis of the study, and led the writing of the paper. ASB, ALB, JFS, and CCV all participated in the development of the questionnaire. KR led the analysis of the study and assisted with the writing of the statistical methods. All authors participated in the design of the study, and provided substantive feedback on the manuscript.
